# Blood-Derived Extracellular Vesicles as a Promising Liquid Biopsy Diagnostic Tool for Early Cancer Detection

**DOI:** 10.3390/biom14070847

**Published:** 2024-07-14

**Authors:** Dan He, Bozhou Cui, Hongkai Lv, Shuxian Lu, Yuan Zhu, Yuqiang Cheng, Lin Dang, Hong Zhang

**Affiliations:** 1 Laboratory of Animal Center, Medical Experiment Center, Shaanxi University of Chinese Medicine, Xianyang 712046, China; hedan069@sntcm.edu.cn (D.H.); 1471027@sntcm.edu.cn (S.L.); 1471029@sntcm.edu.cn (Y.Z.); 2 Department of Experimental Surgery, Tangdu Hospital, Fourth Military Medical University, Xi’an 710038, China; cuibozhou@fmmu.edu.cn; 3 Department of Clinical Medicine of Second Clinical Medical School, Shaanxi University of Chinese Medicine, Xianyang 712046, China; 522030101512@email.sntcm.edu.cn (H.L.); 522030101510@email.sntcm.edu.cn (Y.C.); 4 Basic Medical Academy, Shaanxi University of Chinese Medicine, Xianyang 712046, China

**Keywords:** blood-derived extracellular vesicles, liquid biopsy, early detection, cancer diagnosis, cancer biomarker

## Abstract

Cancer poses a significant public health challenge worldwide, and timely screening has the potential to mitigate cancer progression and reduce mortality rates. Currently, early identification of most tumors relies on imaging techniques and tissue biopsies. However, the use of low-cost, highly sensitive, non-invasive detection methods for early cancer screening has become more attractive. Extracellular Vesicles (EVs) released by all living cells contain distinctive biological components, such as nucleic acids, proteins, and lipids. These vesicles play crucial roles in the tumor microenvironment and intercellular communication during tumor progression, rendering liquid biopsy a particularly suitable method for diagnosis. Nevertheless, challenges related to purification methods and validation of efficacy currently hinder its widespread clinical implementation. These limitations underscore the importance of refining isolation techniques and conducting comprehensive investigations on EVs. This study seeks to evaluate the potential of liquid biopsy utilizing blood-derived EVs as a practical, cost-effective, and secure approach for early cancer detection.

## 1. Introduction

According to the National Center for Health Statistics, nearly 1670 cancer-related deaths were reported daily in the United States by 2023, which already is a major public health concern [1], driven most significantly by delays in diagnosis. Therefore, finding biomarkers for early cancer screening in the population is of great significance. Despite advances in cancer-screening tests that have increased 5-year survival rates from 49% in 2012 to 68% in 2018 [1,2], no study has conducted high-quality screening for early cancer. High-quality cancer screening not only effectively assesses the tumor type, stage, and grade but also balances the risks of overdiagnosis and delayed cancer diagnosis to reduce the incidence of poor prognosis. Additionally, the measure and the cost of a screening test should be acceptable to the population [3], and it should have four main characteristics, namely reliability (instrument consistency and temporal stability), validity (consistency in stability of results), sensitivity (correct case classification), and specificity (identification of disease types or noncases). Common cancer screening typically includes a physical examination, pap smears, imaging studies, and genetic testing. Although the initial cancer probability assessment includes physical and genetic testing, tissue biopsies and imaging are more accurate. However, the high cost and invasiveness of both limit their effectiveness [4]. With the continuous attempts of liquid biopsy in cancer diagnosis, it may be reasonable to believe that in the near future, this method will effectively avoid these disadvantages and will be more convenient, economical, and safe.

**Figure 1:**
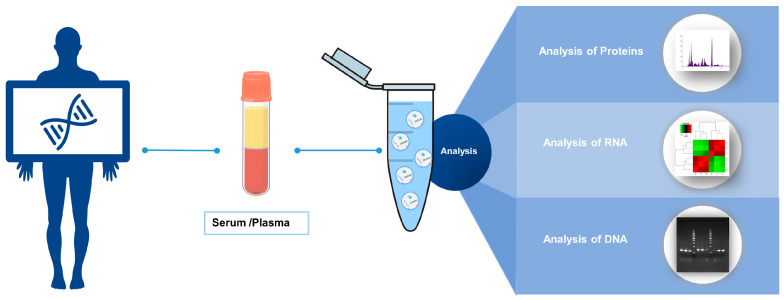
The scheme of blood-derived EVs as liquid biopsy biomarker. EVs were isolated from the peripheral blood of patients, and then the substances they contained were identified to determine the type and grade of the tumor.

Like saliva, urine, and sweat, blood is easier to obtain from patients than cerebrospinal fluid and is rich in various biochemical macromolecules that can be detected by a variety of laboratory tests. Therefore, blood has the advantage of being a source of early diagnostic markers for cancer [5,6]. Circulating tumor DNA (ctDNA), circulating tumor cells (CTCs), and EVs in peripheral blood are gradually favored by researchers, but the former two are difficult to use as candidate markers for early tumor screening because of their undetectably low levels in humans and the difficulty of separation technology. EVs, referring to particles separated by a phospholipid bilayer that are released from the cell and cannot replicate on their own [7], have become the focus of researchers due to their characteristics such as being secreted by all human cells, being present in all biological fluids, and having a specific payload and stable structure. EVs enter the intercellular space after being produced from the plasma membrane and spread throughout the body through lymphatic circulation or blood circulation. EVs can carry complex biomolecules, transmit cellular information, and mediate long-distance communication in physiological processes or pathological states [8,9]. Many pathological processes are mediated by EVs, especially in tumors [10,11]. The contents enriched in EVs will be taken up by nearby or distant recipient cells, further promoting cancer growth and metastasis [12,13]. Furthermore, EVs can dispose of ECM components to alter matrix composition and sclerosis in a variety of ways, while the ECM can also stimulate cancer cells to release EVs, thereby promoting tumor progression through the microenvironment [14]. Related research has proved that EVs produced by tumor cells have biomolecules with parental-specific imprinting information that can support their better survival and development [15]. In addition, the number of EVs isolated from the blood of cancer patients was significantly higher than that of healthy subjects, and EVs carried markers reflecting their cell origin [16]. Based on the above, the use of blood-derived EVs as a liquid biopsy tool has become a promising research direction for early tumor diagnosis (Figure 1).

This review discusses the discovery history, classification, production, and characteristics of cancer-cell-derived EVs and illustrates the advantages of humoral blood-derived EVs as a tool for early tumor humoral biopsy. Furthermore, we summarize the progress in research on EV cargos, particularly proteins and RNAs, from the peripheral blood of cancer patients as early tumor diagnostic markers in the past 3 years. At the same time, the various EV isolation methods used are detailed, further detailing their limitations. Finally, this review summarizes the current challenges of using EVs for early tumor diagnosis via body fluid biopsies. This review aims to provide comprehensive information to support the use of blood-derived EVs as an accurate and effective method for early tumor screening in body fluids.

## 2. Discovery History of EVs

The study of EVs has roots dating to the 1950s, when British scientist Peter Wolf first observed and described small particles, known as platelet dust, originating from platelets via high-speed centrifugation and electron microscopy [17]. Subsequently, in 1971, Neville Crawford isolated similar small particles from platelet-free plasma, termed microparticles, and demonstrated their cargo composition comprising lipids, ATP, and contractile proteins [18]. This discovery suggests that these microparticles may possess biological capabilities. Furthermore, contemporaneous research reported EV-like structures via electron microscopy. Sun identified vesicular structures within the alveolar cavity, potentially originating from alveolar cells [19]. H. Clarke Anderson observed vesicles of varying sizes within the hypertrophic cartilage matrix, indicating a potential role in bone mineralization [20]. During the 1980s, Johnstone and Pan identified the ability of reticulocytes to release peptide-containing vesicles, termed “exosomes”, during in vitro maturation [21,22]. These exosomes are believed to eliminate cellular metabolic waste, including degraded proteins [23]. This groundbreaking finding has since generated significant interest among researchers in the EV field, establishing it as a prominent area of study within the scientific community. Between 2000 and 2010, over 4000 academic papers on EVs were published, culminating in the inaugural International Conference on EVs in Montreal in 2005 [24]. The establishment of the International Society for Extracellular Vesicles (ISEV) six years later further facilitated the dissemination of leading research findings in the EV field and standardized nomenclature associated with EVs. As research on EV advances, scholars have increasingly turned their focus towards exploring the potential clinical utility of the biomolecules present in body fluid EVs, such as proteins, nucleic acids, and lipids, for diagnosing a range of diseases.

## 3. Heterogeneity and Features of Tumor-Derived Extracellular Vesicles

Due to the nanoscale size and heterogeneity of EVs, isolating highly purified EVs presents one of the challenges [25,26]. These particles not only differ in size and biophysical properties but also contain distinct contents, suggesting that these subclasses may serve vital functions in cancer. Firstly, this section describes the classification, biogenesis, and characteristics of tumor-derived EVs are described.

### 3.1. Classification of EVs

**Figure 2:**
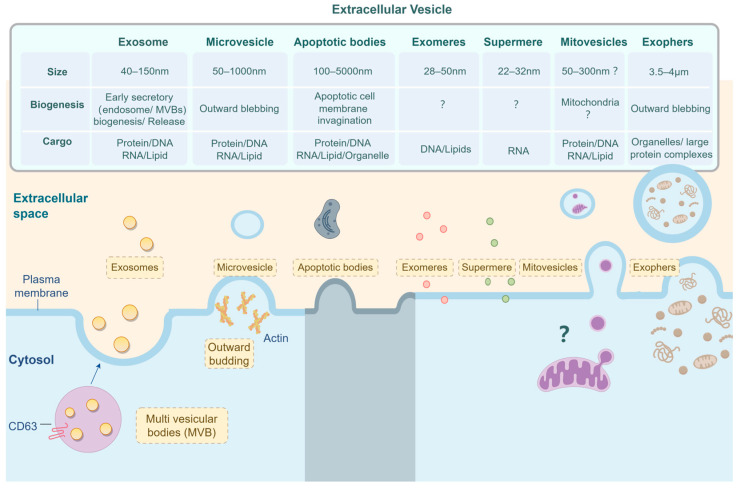
Presentation of EV subtypes. EVs are secreted to extracellular space by cells, and they are classified into seven subtypes in this review: exosomes, microvesicles, apoptotic bodies, exomeres, supermeres, mitovesicles, and exophers.

Based on their physical size, biochemical characteristics, and cellular origin, EVs can be classified into three main subtypes: exosomes (40–150 nm), microvesicles (50–1000 nm), and apoptotic bodies (100–5000 nm) (Figure 2) [27].

Advances in isolation and identification have revealed the discovery of EV isoforms with smaller diameters, including exomeres (28–50 nm) and supermeres (22–32 nm) [25,28,29]. Exomeres are characterized by the absence of a membrane bilayer containing a high amount of DNA and lower levels of lipids [30]. Conversely, the contents of supermeres are somewhat different from those of other microvesicles, exhibiting enhanced uptake compared to other nanovesicles [31]. A significant number of extracellular RNAs have been identified in supermeres rather than in exosomes, with tumor-derived supermeres showing a strong correlation with tumor metabolism and drug resistance [29]. The production processes and biological roles of exomeres and supermeres remain subjects of ongoing research.

Recently, a variety of vesicles with distinct functions have been documented. In a study conducted in 2021, D’acunzo et al. utilized high-resolution density gradient separation to identify a type of double-membraned EV lacking exosomal markers and closely linked to the mitochondria, termed ‘mitovesicles’ [32]. As mitovesicles have only recently been characterized and studied, their role in tumorigenesis remains ambiguous. Given the critical role of mitochondria in energy production in cancer cells, it is possible to suggest that mitovesicles play an important role in tumor metabolism and progression. Rapid progress in isolation technology in recent years has enabled the identification and isolation of more micro-sized vesicles. Exophers are among these vesicles, which are large vesicles measuring 3.5–4 μm found in neurons of the cryptic rod nematode C. elegans. Exophers contain organelles, large protein complexes, and other components, and they play a role in the clearance of damaged, degraded, or aggregated material as well as dysfunctional mitochondria. Studies have demonstrated that exophers play a crucial role in neurological pathologies, as evidenced by research conducted by Melentijevic et al. (2017) and Arnold et al. (2023) [33,34]. Limited evidence is available regarding the involvement of exophers in tumors. However, due to the significant role of exophers containing protein polymers and organelles in cellular processes, it is postulated that exophers are closely related to the rapid progression of tumors.

The overlapping sizes of various particles complicate their comprehensive examination, requiring the development of more precise and effective separation and analysis methods. While progress has been made, such as the classification of small EV subsets from human dendritic cells by Jeppesen et al. into exosome and non-exosome categories, the complexity of EV biogenesis, size, and density presents challenges in achieving a more refined classification [35].

### 3.2. Biogenesis of EVs

Exosome biogenesis begins with the sorting of early endosomes via endocytosis, followed by late endosome sorting mechanisms. Subsequently, intracellular multivesicular bodies (MVBs) containing intraluminal vesicles (ILVs) are generated. These ILVs are released either by fusing the MVB’s outer membrane with the cell membrane or through degradation by lysosomes or autophagosomes. Traditional electron microscopy techniques reduce the size of exosomes, resulting in a cup-shaped morphology, which is considered an artifact but is commonly used as a marker for identifying exosomes [36]. In addition, exosomes can be identified by typical markers such as CD9, CD81, CD63, TSG101, and Alix. Microvesicles are produced through the outward budding of the cell membrane, involving intricate molecular rearrangements that facilitate the formation of smooth vesicles by aiding in membrane bending and potentially reorganizing actin [37]. Apoptotic bodies, as described by Cotter et al. (1992) [38], are EVs involved in cellular remodeling that contain a variety of components and are subsequently phagocytosed. Understanding of the mechanisms underlying the biogenesis of EVs, particularly exosomes, has improved in recent years. It remains unclear whether the pathways of EVs are altered in cancer cells and if tumor cells exhibit a preference for specific pathways. The implications of pathway selection by tumor cells on their survival relative to non-tumor cells have yet to be definitively determined. However, research has been conducted on certain proteins within the pathway in tumors or cell lines, demonstrating their role in tumor metabolism and progression. Exosome biogenesis requires these endosomal sorting complexes for the transport (ESCRT) pathway to facilitate the remodeling of membranes to enclose ILVs within membrane-bound bodies (MVBs). Vps4, an essential factor of this pathway, has been shown to be dysregulated in a variety of tumor types, suggesting it may promote cancer stem cell migration by inducting exosome formation [39,40,41]. Furthermore, the inactivation of Vps4b has been shown to enhance the susceptibility of pancreatic cancer cells to T-cell-mediated cytotoxicity, leading to the stimulation of exosome production [42]. Additionally, ALG-2-Interacting Protein X (ALIX) has been found to interact with Vps4 to facilitate the formation of ILVs within MVBs in non-small-cell lung cancer. This interaction results in the packaging of circTLCD4-RWDD3 into exosomes through the recruitment of ESCRT-III, promoting lymph angiogenesis and lymph node metastasis [43]. The regulation of invadopodium formation via the Rac1-dependent pathway may also play a role in the exosome biogenesis process in cancer cells [44,45]. The activation of Rac1 has been demonstrated to facilitate the progression of cancer, potentially leading to an increase in exosome biogenesis. Furthermore, the upregulation or heightened activation of other oncogenic proteins, such as RAS or EGFR, which are implicated in exosome formation, may augment exosome secretion or alter the composition of exosomal cargo. This phenomenon is supported by multiple previous lines of evidence [46,47,48,49]. The presence of exosomes in the blood of cancer patients has been found to be higher than that of healthy people, suggesting that exosome secretion is closely linked to tumor development [50,51,52,53]. It remains unclear whether tumorigenesis actively promotes exosome production and what distinct characteristics differentiate tumor exosome generation from that of normal cells. Further investigation is warranted to elucidate these inquiries.

### 3.3. Features of Tumor-Derived EVs

**Figure 3:**
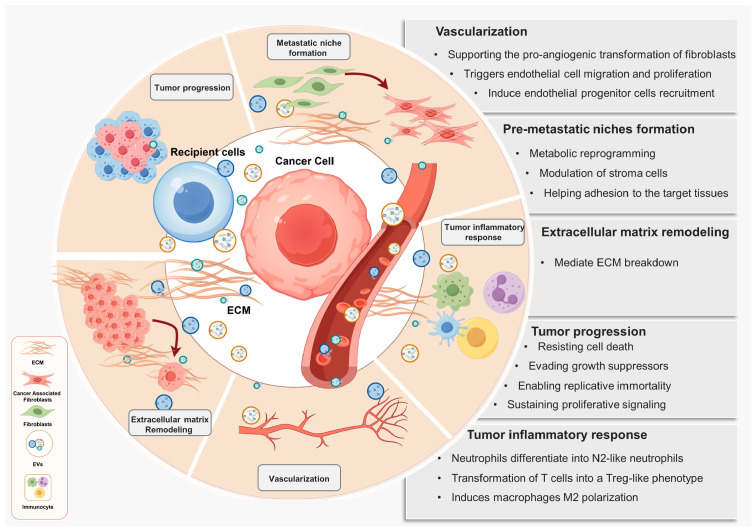
The biological effects of tumor-derived EVs. These EVs influence various biological processes such as tissue extracellular matrix remodeling, vascularization, metastatic niche formation, and tumor inflammatory response.

Tumors thrive in a hypoxic, acidic environment compared to normal tissue cells, leading to persistent inflammation. This condition facilitates EV production in tumors, which plays an extremely important functional role in microenvironmental and signaling pathways. Tumor-derived EVs transport various biomolecules, including proteins, DNA, and ncRNA, to both the tumor microenvironment and nonmalignant cells (Figure 2). This information has implications for extracellular matrix remodeling, vascularization, metastatic niche formation, and tumor inflammatory response, all of which play an important role in the growth rate, invasion, and drug resistance of tumors [54,55,56] (Figure 3). Specifically, the identification of pIgR in serum EVs of hepatocellular carcinoma (HCC) patients is linked to enhancing cancer stemness in HCC cells, thereby contributing to the initiation and progression of liver cancer [57]. Melanoma cells release a significant quantity of EVs containing nerve growth factor receptors, which are taken up by lymphoid endothelial cells through the lymphocyte system and affect the premetastatic microenvironment. This phenomenon stimulates lymph angiogenesis and expedites tumor metastasis [58]. Additionally, EVs derived from hypoxic glioma stem cells, carrying miR-30b-3p, confer temozolomide resistance in glioblastoma [56].

## 4. Advantages of Blood-Derived EVs as a Biomarker Source in Cancer Diagnosis

**Figure 4:**
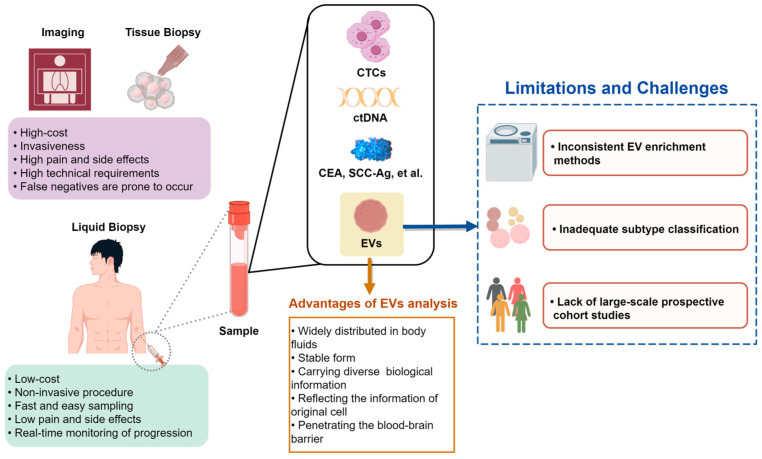
The advantages and limitations of using blood-derived EVs in body fluid biopsy for early tumor screening. In comparison to tissue biopsy, liquid biopsy offers advantages such as being low-cost, non-invasive, and easy to acquire; having minimal side effects; and allowing real-time monitoring of tumor progression. Compared to CTCs, ctDNA, CEA, and SCC-Ag in blood, EVs exhibit characteristics such as widespread distribution, stable form, carrying different biological information, reflecting the information of original cells, and blood–brain barrier permeability. However, the utilization of blood-derived EV cargo as early tumor screening markers is constrained by various factors such as inconsistent EV enrichment methods, inadequate subtype classification, and a dearth of large-scale prospective cohort studies.

Currently, imaging and biopsy serve as the primary modalities for early tumor detection. However, imaging techniques are often costly and financially burdensome for patients, often failing to definitively confirm the early stage of tumor diagnosis. For instance, mammography is the sole clinically validated imaging tool for early detection of breast cancer, yet it exhibits high rates of false negative results, limited sensitivity in dense breast tissue, and a significant number of missed diagnoses. It is common to find that lymph node or systemic metastasis has occurred when a patient decides to undergo a biopsy for diagnosis. Similar to breast cancer, the majority of tumors necessitate an additional tissue biopsy for definitive diagnosis. However, the invasiveness and high risk of tumor tissue biopsy limit the application of this approach. Furthermore, the molecular and genetic data derived from biopsy procedures offer limited early diagnostic insight for early detection, resulting in a low benefit rate and consequently reduced poor patient reliance. Additionally, the impracticality of conducting repeated biopsies on potentially cancerous tissue for ongoing tumor monitoring also poses significant challenges in clinical practice. If the biopsy material is obtained from an atypical site, it could potentially impact the subsequent diagnostic outcomes of molecular and immunological analyses, leading to false negative results or an underestimation of the tumor’s stage of progression. Due to these factors, imaging and biopsy are presently deemed unsuitable for diagnostic or screening purposes in large populations. In response to the technical obstacles associated with consistently identifying and validating early tumor markers, liquid biopsy has emerged as a promising alternative. There are many sources of liquid biopsy; among them, cerebrospinal fluid has a high complexity, and its strong invasiveness and discomfort for patients impede its use as a screening method for the general population. Blood, like saliva and urine, is simple to operate and easy to collect in an outpatient clinic. Blood is rich in a variety of biomolecules that can be tested by a variety of laboratory methods, making it an excellent source of diagnostic markers. The biomarkers in blood include circulating tumor DNA (ctDNA), circulating tumor cells (CTCs), circulating tumor markers, and EVs [59,60,61]. Among them, CTCs and ctDNA have been investigated as potential biomarkers for tumor diagnosis and prognosis. However, both encounter numerous unresolved challenges [62,63], including the low concentration of CTCs in bodily fluids (1 CTC per 1 billion blood cells) and the heterogeneous nature of CTCs. Furthermore, there is a deficiency in efficient techniques for isolating, identifying, and characterizing CTCs, as well as uncertainty regarding the mechanisms by which CTCs are released into the peripheral circulation. In addition, circulating CTCs have a short lifespan of only 6–30 min, which makes them hard to harvest [64,65,66]. ctDNA faces the same problems, such as short half-life, low concentration, and more complex isolation methods [67,68]. Despite the existence of blood tumor biomarkers such as CEA and SCC-Ag, their utility in the early diagnosis and detection of tumors is limited. For instance, CEA, commonly utilized as a tumor marker in gastrointestinal tumors, typically exhibits elevated concentrations in the late stages of tumors rather than in the early stages [69]. Similarly, alterations in SCC-Ag levels are predominantly observed in the advanced stages of non-small-cell lung cancer, rendering it unsuitable as a marker for early tumor diagnosis [70]. As a result, a significant number of researchers have shifted their focus towards blood-derived EVs as potential biomarkers in body fluid biopsies for the purpose of identifying biomarkers suitable for the early detection of tumors. EVs are generated by nearly all human cells, including early tumor origin, and are subsequently released into the interstitial space and widely dispersed throughout various body fluids, including blood, urine, saliva, bronchial, alveolar fluid, breast milk, and fecal supernatant. The diverse origins of EVs in body fluid samples offer increased convenience for diagnostic purposes, enabling real-time monitoring with minimal patient discomfort and high patient compliance [71,72]. Although the clearance mechanism and half-life of blood EVs are still inconclusive [73], it is recognized that EVs have certain stability and low immunogenicity [74] and can protect their cargo for long-distance and stable transportation. In addition, tumor cells may release more EVs into body fluids compared to normal cells. For instance, an analysis of circulating EVs in glioblastoma patients has revealed that a substantial proportion originates from cancer cells, providing a quantitative advantage for utilizing blood-derived EVs in the diagnosis of tumor staging, grading, and typing [75]. Furthermore, the cargo of blood-derived EVs is abundant in tumor-specific information, including cell surface markers and oncogenes indicative of their cellular origin. EVs have shown promise in the early detection of tumors and in assessing tumor heterogeneity, invasion, and metastasis. Given these advantages, researchers are actively investigating the potential of blood-derived EVs as a valuable tool for early liquid biopsy in tumor detection (Figure 4).

## 5. Progress in Research on Blood-Derived EV Biomarkers for Early Cancer Diagnosis

### 5.1. The Candidate Proteins in Blood-Derived EVs

Tumor cells can assemble specific proteins into EVs, including nuclear proteins, cytoplasmic proteins, and membrane proteins. These proteins also contain some specific molecules related to tumorigenesis, which may make EVs a powerful tool for diagnosis and prognosis. Researchers analyzed EVs of 60 cancer cells and identified over 6000 unique proteins, as well as 213 common proteins related to tumorigenesis, including Rabs [76]. Upon comprehensive examination, researchers found that EVs from different cancer types exhibited similar proteomic profiles and formed distinct clusters. Subsequent investigation into a particular cancer type revealed that EV proteins also formed clusters based on the disease stage, suggesting their potential utility as cancer biomarkers. The differential distribution of these tumor-derived EV proteins is commonly attributed to both the inherent intracellular protein levels within the originating cells and the selective enrichment of proteins within the EVs.

Researchers aim to investigate potential variances in EV proteins between individuals with tumors and healthy individuals. The significance of EV proteins as tumor markers was not fully recognized until the emergence of a significant study. Hoshino et al. conducted a comprehensive analysis of numerous human-derived EVs and discovered distinct protein profiles in tumor-derived EVs compared to non-tumor-derived EVs, with certain cancers showing enrichment of specific EV proteins [30]. Moreover, certain proteins have been identified as shared among different types of cancers. Tumor-tissue-derived EVs are mainly secreted by tumor cells, which can reflect the tumor microenvironment and the physiological state of tumor cells [77], and have the ability to enter the peripheral blood plasma. It has been found that plasma-derived EVs and tissue-derived EVs from lung cancer and pancreatic cancer patients share common protein markers, indicating their respective cancer cell origin [30]. This suggests that plasma EVs have a potential correlation with tissue-derived EVs and that the physiological state of primary tumor cells may be directly reflected through easily accessible plasma EVs to a certain extent.

Furthermore, Hoshino et al. conducted an analysis of the plasma EV proteomics in 16 different types of cancer, identifying the abnormal expression of proteins such as immunoglobulin. These findings have the potential to differentiate between cancer and healthy individuals, as well as different types of cancer, with a sensitivity and specificity of 95% and 90%, respectively. The plasma EV protein profile holds promise for future utilization in the early detection and screening of cancer within a broader population. A recent study identified several proteins from serum-derived EVs that can effectively differentiate between patients with primary sclerosing cholangitis (PSC) at risk of developing cholangiocarcinoma. These proteins were used for early potential tumor diagnosis and prevention and to assess patients with cholangiocarcinoma [78]. Additionally, compared to non-cancerous patients, significantly higher type-A receptor 2 (EphA2) levels were noted in serum exosomes of pancreatic cancer patients [79]. Developmental endothelial locus-1 (Del-1) levels in blood EVs were higher in early-stage breast cancer patients than in those with non-cancerous breast diseases [80], while the CD47 level was lower than that of healthy controls [81]. As the isolation technology for EVs in blood has become more widespread and research on cancer cohorts has increased, EV-derived proteins have been found in lung cancer, gastric cancer, breast cancer, prostate cancer, ovarian cancer, and liver cancer [82,83,84]. These proteins exhibit high sensitivity and specificity. More information on representative studies is presented in Table 1. In this study, we summarize EV proteins from the most recent 3 years in Table 1. Although many studies have been conducted on the use of EV-derived proteins as early humoral biopsy diagnostic tools for tumors, there are still some urgent problems to be solved, such as limited blood that can be collected in clinical practice, and EV proteins cannot be obtained in sufficient amounts. In most cases, the target proteins can only be detected using ELISA. However, ELISA requires professional technicians to operate, and its results are susceptible to the influence of temperature, time, and operating level. In addition, the current ELISA detection methods and the chemicals used in the detection have not been internationally standardized, which may make the results relatively unstable, slightly less reproducible, and prone to false positives or false negatives, thus being misdiagnosed or missed [85].

### 5.2. The Candidate Nucleic Acids in Blood-Derived EVs

In addition to proteins, EVs contain many RNA transcripts (oncogenes and tumor suppressor genes), DNA fragments with mutation sites, and ncRNAs (microRNAs, lncRNAs, and circular RNA). Several EV contents are highly disease-specific and may be novel cancer biomarker sources.

DNA derived from circulating tumor EVs has been discovered over the past decade, and the role of DNA diagnostic markers has aroused the interest of researchers. The amount of tumor-cell-derived EV DNA was 20-fold higher than that of fibroblast-derived EV DNA [95]. Therefore, high plasma EV-DNA mutation rates are more effective than those in tissue sample DNA [96]. EV-DNA mutations in biofluids can be accurately detected in glioblastoma cells, allowing for tumor classification [97]. The role of EVs as a future melanoma biomarker is supported by mutant allele frequency DNA detection extracted from patient plasma DNA [98]. Additionally, abundant epidermal growth factor receptor (EGFR) mutations have been detected in EVs isolated from the serum or bronchoalveolar lavage fluid of lung cancer patients [99,100]. Furthermore, DNA methylation differences among CTC metastatic castration-resistant prostate cancer revealed that glutathione s-transferase pi 1 and Ras association domain-containing protein 1A methylation in EVs were correlated with overall survival [101]. The EV DNA methylome enables non-invasive brain tumor classification [97]. These findings demonstrate that DNA effectively identifies and grades tumors, but its ability is limited in identifying early-stage tumors [98,102,103]. Detection is currently being performed using urine and blood for early EV RNA diagnosis. EVs contain mixed RNA, mainly microRNAs, circRNAs, lncRNAs, and mRNA [104].

With the in-depth study of the tumorigenic effect of EV-derived RNA (EVRNA), the role of EVRNA in promoting tumor proliferation, angiogenesis, metastasis, and drug resistance [105] has been gradually recognized. Next-generation sequencing technologies and stable plasma EVRNA levels allow for the sequencing and profiling of minimal EVRNA [106]. EVRNAs have demonstrated high tumor-type-specific accuracy, particularly in early cancer liquid biopsies [107,108]. For example, mutant androgen receptor variants 7 and 9 have been detected as circulating plasma EVRNA tumor biomarkers in prostate cancer patients [109]. Additionally, specific microRNAs, lncRNAs, and circRNAs have recently been reported in plasma or urine [110,111,112]. EV-long RNA methylsterol monooxygenase 1 from patient plasma can identify early breast cancer [113]. Compared to patients with benign nodules, miR-520c-3p and miR-1274b levels are higher in early-stage non-small-cell lung cancer patients and steadily increase throughout the cancer process [113].

Furthermore, specific RNA was identified using EV-derived body fluid isolated from many other tumor types (cholangiocarcinoma, ovarian, hepatocellular, endometrial, and gastrointestinal cancers) [14,67,114,115].

Several bio-companies have performed clinical trials to evaluate and develop this technology. Related tumor marker research is constantly updated as basic research, large-scale clinical cohorts, and EV extraction technology advance. Thus, new research results may replace currently known tumor candidate markers. Marker cohorts have been utilized to screen different tumors, improving tumor identification and missed detection rates. Representative studies are summarized in Table 2. Although some specific RNAs have good sensitivity and specificity, it is necessary to further verify the concise, effective, and accurate marker combinations across patients of different regions, races, and ages [67,116,117]. RNA combinations can comprehensively assess the probability of tumor occurrence from more perspectives, thereby reducing the possibility of misdiagnosis. The screening of signature RNA combinations and the development of clinical diagnostic kits may be a trend for future development. 

### 5.3. Extraction and Quality Control Methods of EVs

Although there have been many studies on the use of EV biomarkers in blood for early tumor diagnosis, poor reproducibility, mainly caused by the heterogeneity of EV isolation and extraction methods, is an inevitable problem. Aan Deun et al. summarized previous studies related to EV isolation and found that there were 1038 isolation protocols and 190 isolation methods [138]. This methodological heterogeneity, coupled with inadequate reporting of laboratory protocols, poses significant challenges in data interpretation and hinders the establishment of robust scientific conclusions. Therefore, the standardization, rapidity, and low cost of EV isolation methods are likely to enable the application of EV-derived diagnostic markers in clinical practice. Rigorous separation strategies are essential for accurate and efficient biochemical EV analysis. Currently, the main challenge is complex body fluid substances, which are often contaminated with other vesicles, soluble proteins, lipids, DNA, and RNA. There is currently no standardized “gold standard”.

Ultracentrifugation is currently the most used laboratory method [108,139], and its EV analysis results are more reliable than those of other methods, as no additional reagents or chemicals are used in the separation. However, this extraction method is complex, time-consuming, and difficult to operate, which is not conducive to large-scale clinical screening or generalization. Compared to differential centrifugation, affinity chromatography using monoclonal antibody-coated magnetic beads achieves a higher exosome yield [140]. EVs can be isolated from small sample sizes using antibodies and their surface markers, including CD9, CD63, CD81, and epithelial cell adhesion molecules. Similarly, EV separation could be performed using cellulose membrane affinity from the spinning column and exoEasy kit (Qiagen, Germantown, MD, USA). However, this was often accompanied by protein and lipid contamination [141,142]. The isolation of EVs using affinity has high purity, good specificity, greater EV subset isolation abilities, and more accurate tumor diagnoses. Although antibody coating is costly, large sample volumes do not limit EV production. However, antibody complex formation and EVs make intact EV isolation difficult, which is unsuitable for EVs requiring intact structures [143]. Rapid EV separation method development from numerous samples in a high-throughput manner will significantly improve research and clinical EV applications.

The emergence of microfluidics has solved the prolonged processing time (from 9 to 3 h), low efficiency (25 to 42%), and large sample size requirements (30–50 μL). Additionally, sample separation and analysis can be integrated into a chip device. Various separation techniques can also be combined, including immune-purification, ultrasonic purification, and electrophoresis. Microfluidics can automatically separate EVs while also being easily operated and modified. Microfluidic purification has a high purity and yield [144]. From the clinical application perspective, solving the shear stress problems associated with low flux, high cost, and EV deformation is necessary. Most importantly, many microfluidic devices have not been validated in subsequent cohort studies. Few devices have been applied to small patient cohorts, requiring further development and exploration.

In addition, it is extremely necessary to unify the laboratory sample quality control, processing, purification, identification, and storage [145]. This standardization allows for accurate cross-validation and EV universality as disease biomarkers across different laboratories and sample cohorts, which dramatically accelerates laboratory research results in translation to clinical practice.

## 6. Limitations and Challenges

Despite the potential of blood-derived EVs as a liquid biopsy tool that could potentially supplant traditional tissue biopsies, numerous challenges persist in light of the current state of research (Figure 4).

The principal obstacle facing the utilization of EV cargoes as biomarkers in early liquid biopsy of tumors is poor reproducibility [146], largely attributed to the lack of consensus on EV enrichment methods across different research laboratories. Despite the availability of various classical separation methods, including ultracentrifugation, monoclonal antibody-coated magnetic bead affinity chromatography, and microfluidic technology, these techniques are still limited in their application. Ultracentrifugation is impractical for large-scale population screening due to its complexity and cost. Monoclonal antibody-coated magnetic bead affinity chromatography is susceptible to contamination from other liquid components, and microfluidic technology has yet to be widely adopted. Hence, there remains a need for enhancement in EV extraction technology to increase yield, expedite processing time, and lower costs. Additionally, standardization of EV production procedures is essential to enable laboratories to consistently isolate and purify EVs of high quality using a uniform method. This standardization will facilitate reproducibility in early liquid biopsy of tumors and support subsequent large-scale clinical validation.

Another unresolved problem in this field is the inadequate subtype classification of EVs, which may have different implications for the early detection and prognosis of tumors. Different EV subtypes have heterogeneity in size, volume, and density. However, current relevant studies are often carried out on mixed EV subtypes with different contents. There are few studies on the use of a specific EV subtype for early diagnosis of tumors, so the diagnostic potential of EV subtypes is very worthy of exploration by researchers.

Research conducted between 2000 and 2020 has identified over 30 studies utilizing EVs for diagnostic or therapeutic purposes in clinical trials, with a predominant focus on cancer-related research. Despite the promising findings, the utilization of EV biomarkers in clinical practice remains limited to the trial stage, highlighting the need for further development and validation before widespread implementation. Furthermore, the incorporation of EV diagnostic biomarkers into clinical guidelines is notably scarce. One of the important reasons is that the predictive performance of early screening or diagnosis of EVs mainly depends on the detection value of EV cargo This detection value is influenced not only by the methods used for isolating EVs, but also by variables such as cancer type, tumor location, and metastasis. The variability in this cargo composition can lead to fluctuations in diagnostic results, increasing the likelihood of false positive or false negative outcomes. Furthermore, the clinical validation of EV markers necessitates prospective cohort studies conducted in a randomized population, with subsequent evaluation against tissue biopsy as the gold standard for diagnostic performance assessment. However, the inherent challenges of conducting prospective cohort studies, including time and cost constraints, as well as the need to account for potential confounding factors, complicate the process of identifying EV tumor markers suitable for clinical application. The identification of early EV diagnostic markers for tumors with low prevalence necessitates a significant financial and temporal investment, thereby constraining research funding in this area.

## 7. Discussion

The identification of reliable biomarkers for early cancer screening and timely intervention is essential to improve patient survival and quality of life. The advantages of easy collection of bodily fluids, high patient acceptance, continuous monitoring of disease progression, and absence of bodily burden have rapidly gained recognition in the field of precision medicine [147]. In recent years, liquid-derived EVs have made significant progress in development due to their diversity of information content and potential efficacy in early tumor detection. Research on EV proteins and nucleic acids has become a hot spot in the early diagnosis of tumors. Therefore, this review focuses on the current status of blood-derived EV proteins and nucleic acids in the early diagnosis of tumors. At the same time, variations in existing EV isolation methods create obstacles to the validation of biomarkers in clinical applications, prompting us to investigate the inherent limitations of commonly used EV isolation techniques.

Uniform and precise terminology facilitates the transparency of research results. According to the MISEV 2023 guidelines [7], the use of the term “exosome” is discouraged due to the lack of consensus on specific biomarkers for EV subtypes, and the term should only be used with great caution if its subcellular origin is experimentally demonstrated. This review is mainly a summary and analysis of the published relevant literature and does not strictly identify exosomes according to the guidelines of MISEV, so the terms related to EVs in the original literature are retained.

The isolation and identification of tumor-cell-derived EVs in blood and the verification of their potential as biomarkers are expected to improve tumor risk stratification, promote guideline decision-making, and improve the overall management strategy, which will become an important research direction in tumor diagnosis in the future. The active search for specific membrane proteins on the surface of tumor-derived EVs and immunocapture with specific antibodies may achieve this goal in the future [148,149]. Monitoring of the differential electroactive components in EV cargo from cancer cells and non-cancer cells by disc carbon fiber microelectrodes also suggests new ideas for cancer diagnosis [148]. If tumor-related information can be obtained through low-invasive humoral EVs, it will be like opening a window to understand the early physiological state of tumors through the peripheral circulation, which will herald a new generation of medical care and realize tumor screening and early diagnosis for the general population.

The translation of blood-derived EV biomarkers into clinical practice requires focused attention. At present, although many humoral EV biomarkers of various tumors have been reported, they are basically in the experimental stage, and few biomarkers can be really applied to clinical practice [150,151], which will require later long-term clinical verification and large-scale prospective cohort studies.

Early tumor screening or diagnosis requires the combination of a variety of indicators, including EV cargoes, and the combined diagnostic accuracy is often better than that of a single biomarker, with higher sensitivity [152,153]. In the future, blood-derived EV biomarkers can be combined with cancer-related risk factors and traditional tumor biomarkers to construct artificial intelligence models, which will be verified by subsequent prospective cohorts. This will make it possible to screen and diagnose individualized tumors in the population in a timely manner and eventually successfully create a new model of personalized tailored tumor diagnosis and treatment.

## 8. Permission to Reuse and Copyright

The authors declare that the research was conducted in the absence of any commercial or financial relationships that could be construed as a potential conflict of interest.

## Figures and Tables

**Table 1:** Representative oncological screening biomarker proteins in blood-derived EVs.

Cancer Type	Cargo	Sample	EV Source	The Assay	Results	Cite
Breast Cancer	CCN1	544 patients and 427 healthy controls	plasma	ELISA	Cancer detective sensitivity of 80% and specificity of 99%	[86]
Breast Cancer	UCHL1	10 patients and 25 healthy controls	serum	ELISA	UCHL1 levels in breast cancer patient serum samples higher than healthy donors, *p* < 0.01	[87,88]
Breast Cancer	AnxA2	169 patients and 68 healthy controls	serum	ELISA	AnxA2 levels in breast cancer patient serum samples higher than healthy donors, *p* < 0.0001	[89]
Breast Cancer	CD151	30 patients and 37 healthy controls	serum	Mass tag-based quantitative proteomics/Western Blot	CD151 expression was significantly increased in the TNBC patient-derived exosomes than healthy donors, *p* < 0.05	[90]
Prostate cancer patients	PSMA	82 cancer patients and 28 benign prostatic hyperplasia	plasma	ELISA	Cancer detective sensitivity of 91.7% and specificity of 83.3%	[91]
Prostate cancer patients	STEAP1	121 patients and 55 healthy controls	plasma	Western blot	Cancer detective sensitivity of 100% and specificity of 76.79%	[92]
Lung cancer	PTX3, THBS1, and CD63	28 early-stage lung cancer patients, 23 benign lung disease patients, and 26 healthy controls	plasma	Surface-enhanced Raman spectroscopy	Cancer detective sensitivity of 92.3% and specificity of 100%	[93]
Lung cancer Lung cancer	CD5L	60 patients and 20 healthy controls	serum	Western blot	Cancer detective sensitivity of 92.9% and specificity of 94.1%	[94]
	NFKBIA, NDUFB10, SLC7A7, ARPC5, SEPTIN9, HMGN1, H4C2, and lnc-PLA2G1B-2:3	64 early-stage lung cancer patients, 24 benign pulmonary nodule patients, and 22 healthy controls	plasma	Western blot	Cancer detective sensitivity of 95.8% and specificity of 91.7%	[13]

**Table 2:** Representative oncological screening biomarker RNA in blood-derived EVs.

Cancer Type	Cargo	Sample	EV Source	The Assay	Results	Cite
Breast cancer	miR-142-5p, miR-320a, miR-4433b-5p	31 patients with invasive ductal carcinoma, 16 healthy controls (CT)	serum	quantitative real-time PCR	Cancer detective sensitivity of 93.33% and a specificity of 68.75%	[118]
Breast cancer	miR-532-502 cluster	354 patients with breast cancer and 404 healthy controls	plasma and serum	quantitative real-time PCR	The AUCs were 0.805 (95%CI: 0.702–0.908, D1) for the three-miRNA panel in plasma, and (95%CI: 0.821–0.969, D2) for the five-miRNA panel in serum.	[119]
Breast cancer	let-7b-5p, miR-106a-5p, miR-19a-3p, miR-19b-3p, miR-20a-5p, miR-223-3p, miR-25-3p, miR-425-5p, miR-451a, miR-92a-3p, miR-93-5p, and miR-16-5p	216 patients and 214 healthy controls	serum	quantitative real-time PCR	Cancer detective sensitivity of 96.2% and a specificity of 94.9%	[120]
Gastric Cancer	long noncoding RNA HOTTIP	126 patients and 120 healthy controls	serum	quantitative real-time PCR	Cancer detective sensitivity of 69.8 and specificity of 85.0%	[121]
Gastric Cancer	long noncoding RNA HOXA11-AS	94 patients and 40 healthy controls	serum	quantitative real-time PCR	Cancer detective sensitivity of 78.7 and specificity of 97.8%	[122]
Gastric Cancer	long noncoding RNA PCGEM1	317 patients and 100 healthy controls	plasma	quantitative real-time PCR	Cancer detective sensitivity of 72.9 and specificity of 88.9%	[123]
Gastric Cancer	long noncoding RNA-GC1	607 patients and 219 healthy controls	plasma	quantitative real-time PCR	Cancer detective sensitivity of 88.24% and specificity of 82.29%	[124]
Gastric Cancer	onco-miRNA panel (miR-10a-5p, miR-19b-3p, miR-215-5p, and miR-18a-5p)	43 patients and 43 healthy controls	serum	quantitative real-time PCR	Cancer detective sensitivity of 85%, 76.32%, 84.38%, 83.78%, 82.89% and specificity of 80.43%, 69.39%, 70.37%, 75.51%, 75.26%, respectively	[125]
Gastric Cancer	miR-1290	100 patients and 50 healthy controls	serum	quantitative real-time PCR	Cancer detective sensitivity of 26 and specificity of 90%	[126,127]
Gastric Cancer	miR-129-1	44 patients and 32 healthy controls	serum	quantitative real-time PCR	Cancer detective sensitivity of 84.2 and specificity of 78.9%	[128]
Gastric Cancer	miR-196a	44 patients and 32 healthy controls	serum	quantitative real-time PCR	Cancer detective sensitivity of 89.5 and specificity of 94.7%	[128]
Gastric Cancer	hsa_circ_0000190	104 patients and 104 healthy controls	plasma	quantitative real-time PCR	Cancer detective sensitivity of 71.2 and specificity of 75%	[129]
Gastric Cancer	circRNA panel	194 patients and 94 healthy controls	serum	quantitative real-time PCR	Cancer detective sensitivity of 78% and specificity of 78%	[130]
Prostate cancer patients	miR-424	58 cancer patients and 6 benign prostatic hyperplasia	plasma	quantitative real-time PCR	Metastatic castration-resistant-derived EVs had higher levels of miR-424 compared to normal and primary tumors	[131]
Prostate cancer patients	miR-423-3p	58 cancer patients and 6 benign prostatic hyperplasia	plasma	quantitative real-time PCR	Cancer detective sensitivity of 80.95% and specificity of 82.41%	[132]
Prostate cancer patients	combinations of miR-141, miR-182, miR-200b, and miR-375	31 cancer patients and 31 benign prostatic hyperplasia	serum	quantitative real-time PCR	AUC = 0.923, 95% CI between 0.8620 and 0.9840	[133]
Lung cancer	miR-23a, miR-361, miR-1228, and miR-let7i	31 patients and 21 healthy controls	serum	quantitative real-time PCR	Cancer detective sensitivity of 52% and specificity of 83%	[134]
Lung cancer	microRNA-10b	80 patients and 69 healthy controls	plasma	quantitative real-time PCR	Cancer detective sensitivity of 98.75% and specificity of 98.55%	[135]
Nasopharyngeal carcinoma	circMYC	210 patients and 158 healthy controls	serum	quantitative real-time PCR	Cancer detective sensitivity of 90.24% and specificity of 94.51%	[136]
Pleural mesothelioma	miRNA panel (miR-11400, miR-148a-3p, miR-409-3p)	82 patients and 82 healthy controls	serum	quantitative real-time PCR	Cancer detective sensitivity of 75% and specificity of 70%	[137]
